# Is spinal sagittal alignment of diffuse idiopathic skeletal hyperostosis relevant to thoracolumbar pain? A controlled study

**DOI:** 10.1186/s12891-022-06084-0

**Published:** 2022-12-27

**Authors:** Shengyu Ruan, Xiaoting Song, Xianquan Xu, Fangying Lu, Chiting Yuan, Binhao Zhang, Tao-Hsin Tung, Dun Hong

**Affiliations:** 1 Department of Orthopedics, Taizhou Hospital affiliated to Wenzhou Medical University, Linhai, China; 2 Bone Metabolism and Development Research Center, Enze Medical Center, Taizhou, China; 3 Department of Radiology, Taizhou Hospital affiliated to Wenzhou Medical University, Linhai, China; 4 Department of Clinical Research, Enze Medical Center, Taizhou, China

**Keywords:** diffuse idiopathic skeletal hyperostosis, spinal sagittal alignments, segmental disc angle, lumbar lordosis, thoracolumbar junction, back pain

## Abstract

**Objectives:**

The extension of diffuse idiopathic skeletal hyperostosis (DISH) from the low thoracic spine to the lumbar spine result in adjustment of spinal sagittal alignment in surgical patients. The aim of this study was to investigate changes in sagittal alignment and back pain in the thoracolumbar spine in nonsurgical DISH and control participants selected from a radiological database.

**Methods:**

Participants in the DISH and the control group were selected by searching for “DISH or degenerative changes in the thoracic spine” in the radiology database of Taizhou Hospital between 2018 and 2021 using Resnick and Niwayama’s criteria. The subjects with spinal tumors, previous spinal surgery, vertebral fractures, inflammatory diseases, poor-quality radiographs, or loss of follow-up were excluded. Demographic and clinical characteristics were recorded retrospectively via the hospital information system and telephone follow-up. Segmental disc angles (SDAs), lumbar lordosis (LL), and bridge scores were analyzed using images of three-dimensional CT.

**Results:**

The final participants consisted of 51 individuals with DISH (DISH group) and 102 individuals without DISH (control group). Depending on the presence of thoracolumbar pain, the DISH group was divided into the DISH group with thoracolumbar pain (DISH+Pain) and the DISH group without thoracolumbar pain (DISH-Pain). The LL and SDAs of T11-T12 and T12-L1 were significantly greater in the DISH group than in the control group. In addition, the SDA of L1-L2 was significantly smaller in the DISH+Pain group than in the DISH-Pain group, whereas there was no significant difference in lumbar lordosis between the DISH+Pain group and the DISH-Pain group. The bridge scores in DISH+Pain group was larger in T10-T11 (*p* = 0.01) and L1-L2 (*p* < 0.01) spine segments than those in DISH-Pain group.

**Conclusion:**

The extension of DISH from thoracic to lumbar spine may increase lumbar lordosis and SDAs in the thoracolumbar spine. The DISH patients with more bony bridging and small L1-L2 SDA may be more likely have thoracolumbar pain. Adjustment of sagittal alignment of the spine in the development of DISH may be of clinical importance.

**Supplementary Information:**

The online version contains supplementary material available at 10.1186/s12891-022-06084-0.

## Introduction

Diffuse idiopathic skeletal hyperostosis (DISH), first described by Forestier and Rotes Querol in 1950 under the term “ankylosing bone hypertrophy” [1], is evolving into a common noninflammatory spondyloarthropathy as our population ages [2, 3]. The hyperostotic ossification typically occurs in the lower thoracic spine and then spreads to the lumbar and cervical spine [1, 2, 4]. The most widely accepted criterion of DISH by Resnick et al. in 1976 includes at least four affected contiguous vertebral segments and preservation of disc spaces [5]. To date, the diagnostic classification of DISH is based on radiological images rather than clinical symptoms, and there is still lack of consensus on the criteria [5, 6]. Many people with this DISH may be completely asymptomatic, while the hyperostotic ossification is discovered incidentally in their spine images. In addition, DISH usually starts from the lower thoracic spine and then spreads to the upper thoracic and especially the lumbar spine over time [5, 7], resulting in decreased mobility and even complete ankylosis of the affected spine [5, 8]. However, the currently used criteria do not consider the progressive nature of DISH [6].

Studies have shown that DISH is not an incidental radiographic finding but has clinical correlates [9–11]. The DISH patients often complain of difficulty in bending or limitations in the thoracic spine [7, 9]. Older men with DISH in the community reported more back pain in the past 12 months than those without DISH [10]. In addition, DISH can lead to disability or limitation in physical functioning in older adults [7], such as decreased grip strength or inability to complete 5 chair stands without arm support [9]. DISH is also associated with complicated thoracolumbar fractures and back pain [12, 13]. However, as with low back pain and lumbar degeneration, the correlation between symptomatic DISH and radiological findings is currently undefined [12].

The sagittal alignment of the spine is an interesting area to understand the correlation between DISH symptoms and radiological findings [8, 11]**.** In patients with lumbar spinal stenosis, DISH leads to kyphotic changes in the lumbar and thoracic spine [14]. In addition, DISH patients with lower fused vertebral ends at the lumbar level had significantly decreased lumbar lordosis (LL) [14]. In patients with cervical myelopathy due to cervical ossification of the posterior longitudinal ligament (OPLL) or spondylosis, DISH was related to excessive thoracic spine kyphosis [8]. Moreover, sagittal alignment chance in DISH individuals tends to cause the abnormal posture [11]. Therefore, the change of spinal alignment and DISH symptoms deserve more attention from the health professionals.

DISH extends to lumbar spine may increase the risk of lumbar disc degeneration and further surgical treatment [15]. DISH increased the onset or severity of lumbar stenosis by decreasing the lower mobile segments of the lumbar spine [16]. However, the DISH participants with sagittal alignment analyzed in previous studies were surgical patients, and it is known that many people with DISH may be completely asymptomatic. A general population is needed to determine the relationship between DISH symptoms in the thoracolumbar region and changes in thoracolumbar spine alignment.

In this study, we investigated the correlation of spinal sagittal alignment with thoracolumbar pain by comparing the LL, segmental disc angles (SDAs), and bridge scores in thoracolumbar and lumbar spine in the participants with or without DISH from a radiological database.

## Methods

### Participants

**Fig. 1:**
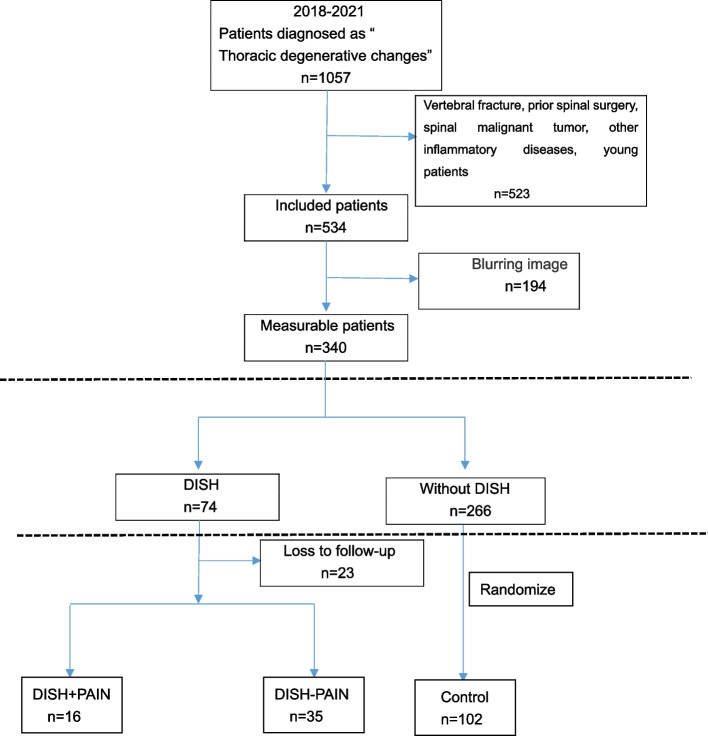
Flow diagram of participant selection

We first screened 1057 consecutive subjects “degenerative changes of thoracic spine or DISH” in radiological reports of three-dimensional CT of thoracic and lumbar spine from Picture Archiving and Communication System (PACS) in Taizhou Hospital between 2018 and 2021. Subsequently, 523 individuals were excluded if they were under 40 years old or had spinal tumors, previous spinal surgery, vertebral fractures, and other inflammatory diseases. In addition, 194 individuals were excluded if the quality of radiographs was not suitable for further radiological evaluation. Of 74 individuals diagnosed with DISH, 51 of them underwent complete followed-up. Of the 266 individuals diagnosed without DISH, 130 individuals were selected by simple random sampling using SPSS software. Of them, 28 individuals were lost to follow-up. Therefore, the remaining 102 individuals who were fully followed up were considered as the control group (Fig. 1). All patients were of Han nationality on the southeast coast of China. The study was approved by the Ethics Committee of Taizhou Hospital of Zhejiang Province (Approval number: K20220507).

### Clinical characteristics

Data on age, gender, BMI, hypertension, diabetes, and back pain were retrospectively recorded from the hospital information system and the presence of thoracolumbar back pain was confirmed by telephone follow-up. In this study, thoracolumbar pain was defined as pain in the middle of back (thoracolumbar region). The main information about thoracolumbar pain recorded in the HIS was the description: ‘pain in the middle of the back, pain in the thoracolumbar region, pain in the upper back or pain in the center of back’. Descriptions of pain in the lumbosacral region and low back pain were not included. Thoracolumbar back pain is further confirmed if there was a description of ‘tenderness in the thoracolumbar region, worsening of symptoms when turning on the bed, or pain in the lower rib area’. In addition, the thoracolumbar pain lasted for at least one month and had occurred in the previous two years.

### Radiological evaluation

**Fig. 2:**
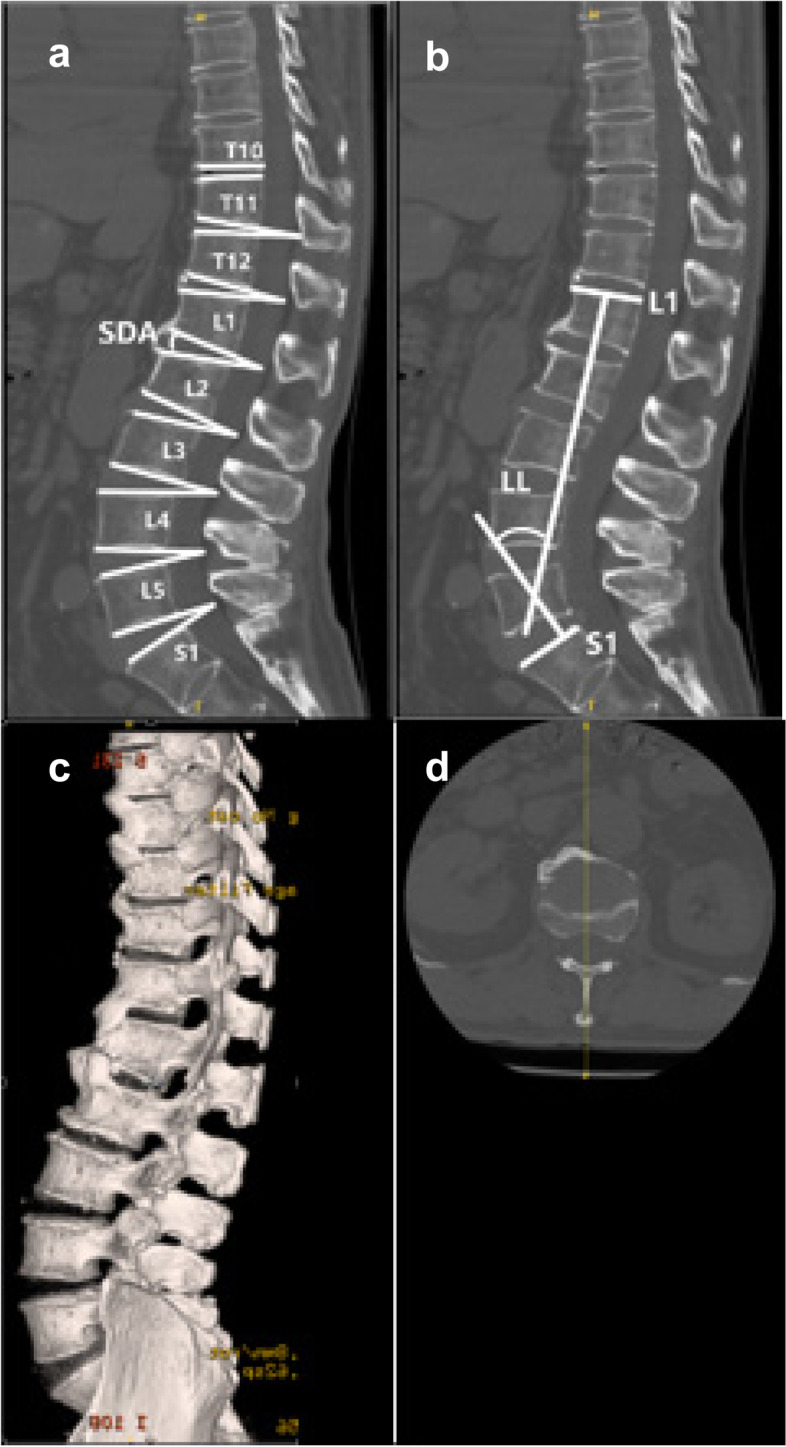
Illustration of the measurement of sagittal parameters in a DISH patient using a three-dimensional CT image. a: Segmental disc angle (SDA) was determined by measuring the angle between the superior and inferior endplates of each disc in the thoracolumbar and lumbar spine; b: Lumbar lordosis (LL) was measured between the superior endplate of L1 and the sacral plate; c: A three-dimensional CT reconstruction image of this DISH participant; d: Sagittal parameters were measured through the center of the spinous processes. Segmental lordosis is signified by positive values, whereas segmental kyphosis is characterized by negative values

**Fig. 3:**
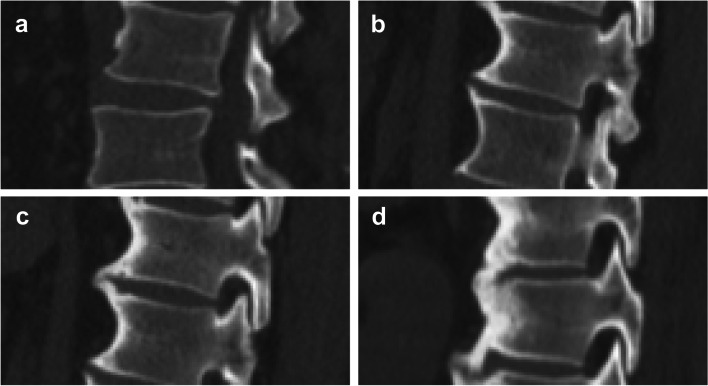
The bridge scores were evaluated in sagittal CT image. A score from 0 to 3 was assigned for each vertebral segment. a: A score of 0 indicates normal vertebral bodies without formation of new bone; b: A score of 1, anterior new bone formation without a solid bony bridge OR a connection between two adjacent vertebral bodies without abundantly formed bone;c: A score of 2, near complete bridging by the anterior new bone formation with less than 2 mm of distance between the bony structures or a full connection of the bone in a maximum of two sagittal or coronal CT sections; d: A score of 3, complete bridging between the vertebral bodies above and below the disc with abundant new bone formation in more than two sagittal or coronal CT section

The spinal parameters included SDAs, which measure the angle between the superior and inferior endplates at each level of the thoracolumbar and lumbar spine (Fig. 2a) and LL, which measures the angle between the superior endplate of L1 and the sacral plate (Fig. 2b). The sagittal parameters on images from three-dimensional CT were evaluated by drawing tangential lines through the center of spinous processes (Fig. 2c and d). The bridge scores were evaluated in sagittal CT images according to the scoring system presented by Kuperus et al. in 2019 (Fig. 3) [17].

Two authors, who were blinded to the patients, diagnosed DISH according to the Resnick and Niwayama’s criteria: “At least four contiguous vertebral segments was affected , sacroiliac joint was not affected, the absence of apophyseal joint ankyloses, and preservation of intervertebral disc spaces” [3]. To verify the reliability of the radiological data, the parameters of twenty randomly selected patients were measured by the two authors twice at a two-months interval. The difference between the measurements of the two authors was not statistically significant (*p* = 0.76).

### Statistical analysis

Participants were divided into the DISH group and the control group according to the presence of DISH. The DISH participants then were also divided into a thoracolumbar pain (DISH+pain) subgroup and no thoracolumbar pain (DISH-Pain) subgroup according to the presence of the thoracolumbar pain. The demographic and clinical characteristics between the DISH and the control group were compared using t-test or chi-square test. The parameters of sagittal alignment measured between the DISH and the control group or between DISH+Pain and DISH-Pain subgroup were compared and analyzed using the group t-test. All data are shown as mean ± SD. A *p*-value of < 0.05 was considered statistically significant. All statistical analyses were performed using GraphPad Prism 8 software (GraphPad Software, San Diego, California, USA).

## Results

### Demographic and clinical characteristics

Mean ages (67.39 vs 65.38 years), percentage of hypertension and diabetes, and gender composition did not differ significantly between the DISH and the control group. The bone mass index (BMI) in the DISH group is significantly higher than that in the control group (Table 1).

**Table 1:** Demographic and clinical characteristics of DISH and control group

Variables	DISH (*n* = 51)	Control (*n* = 102)	*P*-value for t-test or chi-square test
Male	30 (58.82%)	43 (42.15%)	0.05
Age	67.39 ± 11.04	65.38 ± 9.84	0.25
Diabetes	12 (23.53%)	14 (13.73%)	0.13
Hypertension	24 (47.06%)	35 (34.31%)	0.13
BMI	25.40 ± 3.53	23.47 ± 3.093	< 0.01

BMI: Body mass index; Data are shown as the number (percentage) or as the mean ± SD

The mean age, percentage of hypertension, and body mass index (BMI) did not differ significantly between DISH+Pain group and DISH-Pain group. The proportion of men appeard to be smaller and the proportion of diabetics greater in DISH+Pain group than in DISH-Pain group. However, the differences were not statistically significant (*p* > 0.05) (Table 2).

**Table 2:** Demographic and clinical characteristics of DISH + Pain and DISH - Pain group

Variables	DISH + Pain (*n* = 16)	DISH - Pain (*n* = 35)	*P*-value for t-test or chi-square test
Male	8 (50.00%)	22 (62.86%)	0.29
Age	67.50 ± 13.12	67.34 ± 10.16	0.96
Diabetes	6 (37.50%)	6 (17.14%)	0.18
Hypertension	7 (43.75%)	17 (48.57%)	0.52
BMI	25.07 ± 3.92	25.55 ± 3.39	0.66

DISH + Pain: DISH patient with thoracolumbar pain; DISH - Pain: DISH patient without thoracolumbar pain; BMI: Body mass index; Data are shown as the number (percentage) or as the mean ± SD

### Spinal sagittal alignment is altered in DISH participant with thoracolumbar pain

We analyzed the LL and the SDAs between DISH group and control group. The LL of the DISH group was significantly larger than that of the control group (43.79° vs 32.40°) (*p* < 0.01). In addition, the SDAs between T11-T12 and T12-L1 were significantly greater in the DISH group than in the control group (*p* < 0.01 and *p* = 0.02, respectively). However, there was no significant difference in SDAs from L1 to S1 between the two groups (*p* > 0.05) (Table 3).

**Table 3:** Spinal sagittal parameters of thoracolumbar and lumbar spine in DISH and control group

Parameter	DISH (*n* = 51)	Control (*n* = 102)	*P*-value for t-test
Lumbar lordosis (°)			
T10-T11 (°)	43.79 ± 15.30	32.40 ± 14.1	< 0.01
T11-T12 (°)	2.23 ± 3.49	0.78 ± 2.37	< 0.01
T12-L1 (°)	3.50 ± 4.85	1.98 ± 3.06	0.02
L1-L2 (°)	4.23 ± 3.11	3.92 ± 4.04	0.57
L2-L3 (°)	6.57 ± 4.26	6.02 ± 4.30	0.46
L3-L4 (°)	8.61 ± 4.56	7.61 ± 4.09	0.16
L4-L5 (°)	9.38 ± 5.22	8.42 ± 5.62	0.31
L5-S1 (°)	12.18 ± 7.97	11.65 ± 6.89	0.57

Data are shown as the mean ± SD

We also examined the LL and the SDAs between DISH+Pain group and DISH-Pain group. Interestingly, we found that L1-L2 SDA of DISH+Pain group was smaller than DISH-Pain group (*p* = 0.04). However, there were no significant differences in LL and SDAs in other segments between DISH+Pain group and DISH-Pain group (*p* > 0.05) (Table 4).

**Table 4:** Spinal sagittal parameters in thoracolumbar and lumbar spine in DISH + Pain and DISH -Pain group

Parameter	DISH + Pain (*n=*16)	DISH - Pain (*n=*35)	*P*-value for t-test
Lumbar lordosis (°)	21.12±18.74	21.92±18.03	0.80
T10-T11 (°)	1.35±3.06	1.60±3.05	0.79
T11-T12 (°)	2.10±2.88	2.28±3.77	0.86
T12-L1 (°)	3.54±4.69	3.48±4.98	0.96
L1-L2 (°)	2.95±2.28	4.90±3.28	0.04
L2-L3 (°)	6.34±4.94	6.67±4.00	0.81
L3-L4 (°)	8.59±4.26	8.66±4.76	0.96
L4-L5 (°)	8.94±5.06	9.58±5.35	0.69
L5-S1 (°)	14.37±10.21	11.17±6.64	0.19

*DISH + Pain* DISH patient with thoracolumbar pain, *DISH – Pain* DISH patient without thoracolumbar pain, Data are shown as the mean ± SD.

### Osteophyte bridge score is greater in DISH participants with thoracolumbar pain

**Fig. 4:**
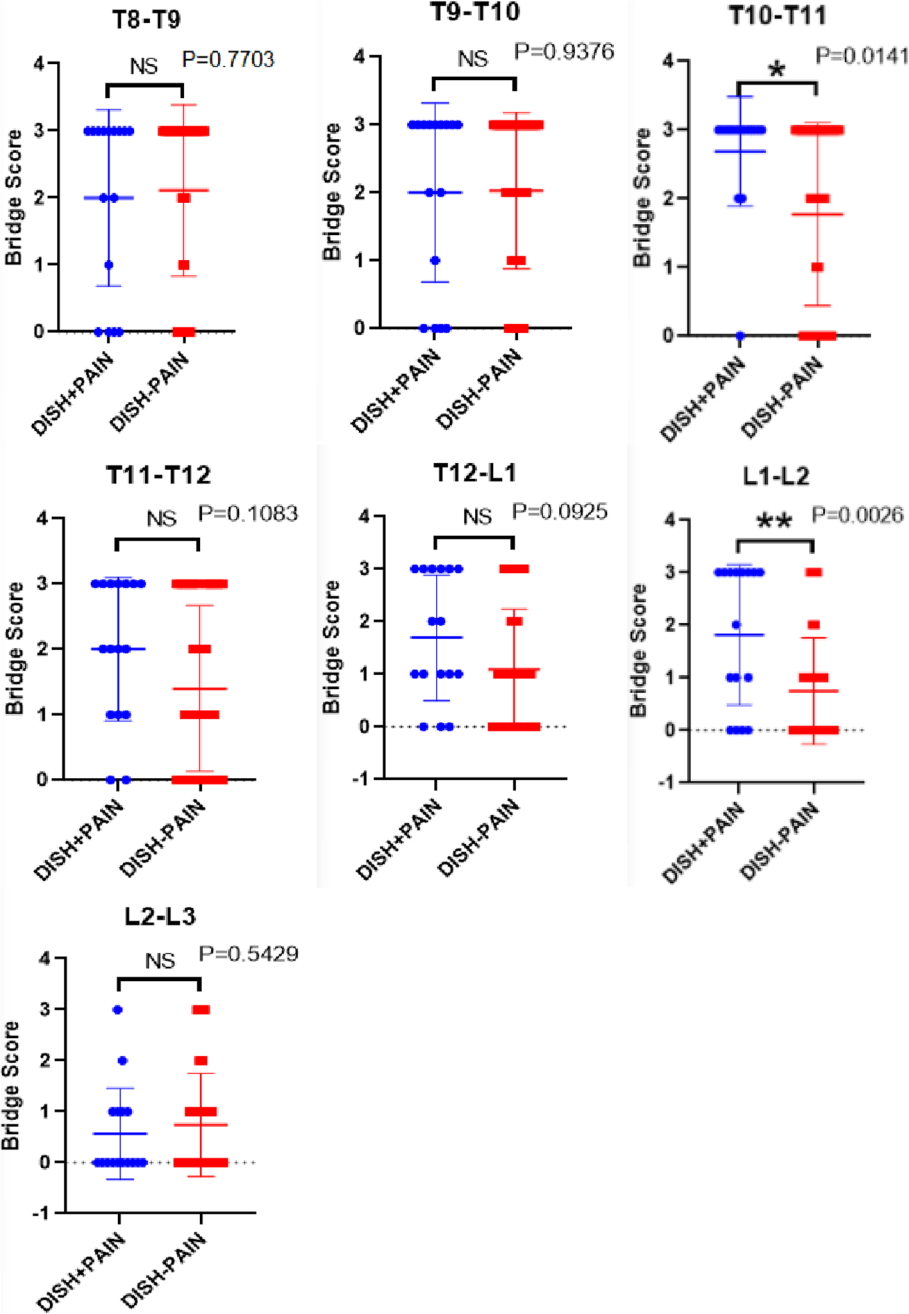
The comparison of osteophyte bridge scores between DISH+Pain group and DISH-Pain group

We measured the osteophyte bridge scores between DISH+Pain group and DISH-Pain group (Fig.4). The bridge scores in DISH+Pain group was larger in T10-T11(*p* = 0.01) and L1-L2(*p* < 0.01) spine segments than those in DISH-Pain group. There were no significant differences in other segments between DISH+Pain group and DISH-Pain group (p > 0.05).

## Discussion

Our data showed that the lumbar lordosis was greater in individuals with DISH. In the study of elderly in rural areas by Uehara et al., the group with DISH had more thoracic kyphosis, but lumbar lordosis was similar to the group without DISH. When the selected subjects were divided into DISH with cervical spine involvement (C- DISH), thoracic spine involvement (T- DISH), or lumbar spine involvement (L- DISH), the DISH with lumbar spine involvement appeared to have a lower LL than DISH without lumbar spine involvement [18]. Previous study also showed that patients in whom DISH extended to lumbar spine demonstrated significantly decreased lumbar lordosis and increased thoracic kyphosis [14]. In contrast to the subjects in that study were from surgical patients with lumbar spinal stenosis (LSS) [14], our study participants were selected from radiologic database, representing individuals diagnosed with DISH by incidental spinal image. Symptomatic LSS usually has a more kyphotic lumbar spine than asymptomatic LSS, which is due to the loss of disc height [19]. In Olivieri’s study, the occiput-to-wall distance increased in patients with very stiff spine similar to advanced ankylosing spondylitis, indicating greater thoracic kyphosis in the late stage of DISH [11]. In addition, DISH in the thoracic spine was associated with a greater Cobb angle (T4 to T12) in both Caucasians and African Americans [20]. In Katzman et al’ s cross-sectionally study, DISH is also associated with greater Cobb angle (T4 to T12) and thoracic kyphosis in older individuals [21]. In patients with cervical myelopathy, patients with DISH are also more likely to have excessive kyphosis in the thoracic spine, a high C7 slope, and a high C2–7 SVA but not in lumbar lordosis [8]. Therefore, the increased LL in our study could be a compensatory postural adjustment for the increasing thoracic kyphosis in the development of DISH.

Our study also showed that SDAs of T11-T12 and T12-L1 were greater in DISH participants. A study on natural course of DISH in the thoracic spine of adult men showed that T8–T9 and T9–T10 formed the most rigid bone bridge in both the pre-DISH (less than four segments) and the DISH group. The fusion scores of T11-T12 and T12-L1 were lower than those of T5-T10 [22]. Using data from whole-spine CT of 1478 Japanese patients who had suffered trauma, Hiyama A et al. found that ossification was commonly locked at T8-T10, and ossification was rarely found in the spine in contact with aorta [23]. The delayed fusion of the two segments may be due to altered mechanical behavior of the thoracolumbar junction [24]. The thoracolumbar junction (T10-L2) connects the rigid rib-bearing thoracic spine to the more flexible lumbar spine and is the area bears greater biomechanical stress and movement than thoracic spine [25]. Therefore, the larger SDAs of T11-T12 and T12-L1 in our study may also be a compensatory adjustment for the more rigid upper thoracic spine.

Surprisingly, DISH patients with smaller L1-L2 SDAs seemed to suffer from thoracolumbar pain, although contrary to our prediction, the data did not support that lumbar lordosis or SDAs of T11-T12 and T12-L1 correlate with thoracolumbar pain in DISH patients. One reason could be that DISH always takes a long-term course [22] and the symptoms may appear later than the radiological images. Solid ossification is easily acquired in a part of the thoracic spine (T5-T10) where the spinal dynamics are limited by the rib cage [22]. Previous research has shown that obese individuals are more likely to develop DISH at a young age (before the age of 50) and complain of lumbar or thoracic spinal pain [26]. However, there are no studies yet on nature evolution of DISH in the thoracolumbar junction where thoracolumbar pain may occur. Our study also showed that the bridge scores were greater in the DISH+Pain group than in the DISH-Pain group in the spine segments of T10-T11 (*p* = 0.01) and L1-L2 (*p* < 0.01), suggesting that the thoracolumbar pain may arise in the process of bony bridging in the thoracolumbar region where DISH extends. At present, mechanical instability cannot explain thoracic spine pain because the thoracic spine is more stable than the lumbar spine and the bone bridge of DISH makes the thoracic spine a solid trunk. However, the thoracolumbar spine is the transition from the thoracic spine to the lumbar spine with high mechanical stress and is usually not firmly fused. Therefore, pain may stem from a stiffer spine with limited motion (more bony bridging and smaller SDA), especially in the thoracolumbar region of DISH. However, longitudinal research of individuals with DISH in the future may be needed to elucidate the development of DISH and pain in the thoracolumbar junction.

At present, there is still no consensus on the classification criteria of DISH [6]. Resnick et al. developed the most accepted criterion in 1976. Researchers tried to stage DISH based on entheseal new bone, number of involved segments, or fusion of pre-discal nucleus and spur. These modified classification criteria were also based on Resnick et al.’s criterion [6, 27, 28]. Therefore, the classic diagnostic classification and staging of DISH is limited only to radiological images without clinical correlation. The awareness of DISH among physicians is still limited [29], unless spinal surgery are needed in DISH patients with traumatic fractures, severe spinal stenosis, or large cervical osteophytes [30, 31]. In this study, we found SDAs in the upper lumbar region, or the thoracolumbar region, may be related to thoracolumbar pain, suggesting that DISH extended to thoracolumbar region may be of clinically significant and predict future clinical symptoms.

Currently, thoracolumbar back pain is defined differently in the literature. R. Maigne described thoracolumbar pain as low back pain of thoracolumbar origin based on clinical features and classic signs such as: localized tenderness over a certain spinous process at the thoracolumbar junction, and tenderness over the affected apophyseal join [32]. In one review, thoracolumbar pain was defined as pain throughout the thoracic, lumbar, and sacral spine, including the inferior gluteal fold and the chest wall from the sternum to the costal region [33]. Thoracic spine pain is also referred to as mid back pain. Mid- back pain (MBP) can be defined as pain in the body region between the 1st and 12th thoracic vertebrae and the corresponding posterior aspect of the trunk [34]. Studies have shown that the incidence of mid-back pain (15–31%) was lower than that of low-back pain (48–67%) in the general population [35]. In our study, the thoracolumbar pain was defined as pain generated from the thoracolumbar region.

This study has several limitations. The radiographs of the three-dimensional CT were taken with patients in the supine position. The CT provides far more detailed imaging of the intervertebral disc spaces and bridging ossifications than regular X-ray [23], and X-ray alone may lead to incomplete diagnosis of DISH for the low quality of images, however, the optional sagittal spine parameters are usually measured when patients are in standing position, and the SDAs may be larger in supine position than standing position. A combination of whole-spine CT and whole spinal standing X-ray may be optimal for measurement of the sagittal spine alignment of DISH. Our controlled study has a limited number of participants and a lack of grading on the pain severity or the quality of life, and the control group was randomly selected without strict matching. In addition, there is no clear delineation between thoracolumbar and low back pain.

In summary, our controlled study shows that individuals with DISH have increased LL and T11-L2 SDAs in the thoracolumbar spine. In addition, the DISH patients with a smaller L1-L2 SDA tended to suffer thoracolumbar pain. We suggest that sagittal alignment of thoracolumbar junction and lumbar spine is adjusted during natural evolution of DISH, which may be of clinical significance.

## Conclusion

The extension of DISH from thoracic to lumbar spine may increase lumbar lordosis and SDAs in the thoracolumbar spine. The DISH patients with early bridge formation and small L1-L2 SDA may be more likely to have thoracolumbar pain. Adjustment of sagittal alignment of the spine in the development of DISH may be of clinical importance.

## Supplementary Information

**Additional file 1.**

**Additional file 2.**

## Data Availability

The datasets used and/or analyzed during the current study available from the corresponding author on reasonable request.
